# Intensive longitudinal modelling predicts diurnal activity of salivary alpha-amylase

**DOI:** 10.1371/journal.pone.0209475

**Published:** 2019-01-23

**Authors:** Jesús F. Rosel, Pilar Jara, Francisco H. Machancoses, Jacinto Pallarés, Pedro Torrente, Sara Puchol, Juan J. Canales

**Affiliations:** 1 Department of Developmental, Educational and Social Psychology, and Methodology, University ‘Jaume I’, Castellón, Spain; 2 Fundación Andaluza Beturia para la Investigación en Salud (FABIS), Huelva, Spain; 3 IATS, Consejo Superior de Investigaciones Científicas, Torre la Sal, Spain; 4 Division of Psychology, School of Medicine, University of Tasmania, Hobart, Australia; Radboud Universiteit, NETHERLANDS

## Abstract

Salivary alpha-amylase (sAA) activity has been widely used in psychological and medical research as a surrogate marker of sympathetic nervous system activation, though its utility remains controversial. The aim of this work was to compare alternative intensive longitudinal models of sAA data: (a) a traditional model, where sAA is a function of hour (hr) and hr squared (*sAA*_*j*,*t*_ *= f(hr*, *hr*^*2*^*)*, and (b) an autoregressive model, where values of sAA are a function of previous values (*sAA*_*j*,*t*_ *= f(sAA*_*j*,*t-1*_, *sAA*_*j*,*t-2*_, *…*, *sAA*_*j*,*t-p*_). Nineteen normal subjects (9 males and 10 females) participated in the experiments and measurements were performed every hr between 9:00 and 21:00 hr. Thus, a total of 13 measurements were obtained per participant. The Napierian logarithm of the enzymatic activity of sAA was analysed. Data showed that a second-order autoregressive (*AR(2)*) model was more parsimonious and fitted better than the traditional multilevel quadratic model. Therefore, sAA follows a process whereby, to forecast its value at any given time, sAA values one and two hr prior to that time (sAA _*j*,*t*_ *= f(SAA*_*j*,*t-1*_, *SAA*_*j*,*t-2*_) are most predictive, thus indicating that sAA has its own inertia, with a “memory” of the two previous hr. These novel findings highlight the relevance of intensive longitudinal models in physiological data analysis and have considerable implications for physiological and biobehavioural research involving sAA measurements and other stress-related biomarkers.

## Introduction

The physiological response to stressful stimuli involves activation of the sympathetic nervous system. Well-recognised biomarkers of such activation include elevated heart rate and blood pressure, pupil dilation and release of glucose from the liver [1]. In recent years there has been growing interest in salivary alpha amylase (sAA) as a potential additional marker of sympathetic nervous system activation. sAA is a digestive enzyme secreted by norepinephrine-responsive salivary gland cells. Activation of the sympathetic nervous system with the beta-adrenergic receptor agonist, isoprenaline, stimulated sAA release [2], whereas its inhibition with the antagonist, propanolol, reduced it [3], suggesting a possible association between sympathetic activity and sAA. Although sAA has been shown to rise in the presence of acute laboratory stressors, including the Trier Social Stress Test [4], and real-world settings, such as academic examinations [5], limitations in its utility in biobehavioral research derive from the fact that local parasympathetic nerves also regulate sAA via (a) release from glands that are exclusively, or in part, parasympathetic, (b) synergistic sympathetic-parasympathetic interaction effects of protein secretion and (c) effects on salivary flow [6]. Despite this caveat, sAA detection could complement data collection of other indicators of the stress response such as plasma catecholamines, blood pressure and heart rate, establishing correlations as required [7].

Previous research indicates that sAA exhibits a distinctive diurnal profile characterised by a sharp decrease after awakening and a steady rise during the course of the day, with variations being mainly associated with chronic stress and mood changes [8]. Such diurnal variations have been explored across the lifespan [9–11] and in a number of clinically-relevant populations [12, 13]. Well-principled statistical prediction and modelling of biological variables such as sAA can provide a useful tool to make decisions informed by participant’s and patient’s physiological and/or clinical history.

The analysis of intensive longitudinal models (ILM) is a process that will become increasingly more frequent in biology, psychology, and medicine due to the improvement of the technology employed in multiple biological, psychological, and medical record systems, and the improvements in statistical analysis procedures that are better suited to the nature of the data [14–16]. This ‘intensive’ data record system, however, receives different denominations, depending on the context in which it is used. Thus, the same type of data is often referred to using different terms (e.g. ILM, panel data, repeated measures, time series, etc.) and processed with different types of data analyses (e.g. panel data, repeated analysis of variance, profile analysis, latent growth analysis, multilevel pooled time series analysis, etc.). Some authors therefore use the term ILM [17, 18] to refer to a situation in which several participants are recorded in different variables at different time points. In other research contexts, mainly in sociology and economics, this kind of research is called ‘panel data’ [19] and involves an identical research measurement procedure: the same participants are measured over time. In a different context, often in experimental research, when the objective is to observe the mean differences of the dependent variables (DVs) as a function of the time or of the longitudinal treatment, an analysis of the variance with repeated measures is frequently used, and functional fitting curves (e.g. linear, quadratic, regression as the trend analysis) are used to predict the DV [20, 21].

Technically, what all these approaches have in common is that the data are ‘pooled time series models’ (PTS), which may be univariate or multivariate [22, 23]. In PTS, the objective is to establish what effect time has on the measured (dependent) variables, and it is possible to detect what effect the same variable has on itself through autoregressive models, where the independent variables (IVs) are past values of the same DV.

Time can be measured in different ways: as the time of day, the age of the participant, time elapsed since the time of the first measurement, and so forth. This type of investigation is used mainly within a context of longitudinal research field studies, where analyses are performed by regression (as a function of time) or by autoregressive models. The advantage of PTS is that it becomes possible to analyse the data with few participants and few points of measurement, as in panel data analysis. It is advisable not to confuse the different terms that refer to ILM with the data analysis used, since the same data can be analysed with different statistical analysis procedures and can receive different denominations. ILM can thus be analysed using different statistical systems, depending on the aims of the research and the researchers’ hypotheses.

The theory regarding the statistical analysis of ILM is firmly established [14, 17, 18, 23–27] and empirical applications of PTS models [28–32]. To test the inner validity of a statistical ILM, it would be necessary to check the residuals. In the present research, we analyse a dataset obtained from a laboratory study in which each participant provided repeated salivary samples such that the content of sAA could be determined. Samples of the saliva from 19 participants were collected every hour (hr), between 9:00 and 21:00 hr on the same day of attendance, this is, a total of 13 measurements were obtained per participant.

The use of autoregressive models is not widespread in the biological and behavioural sciences where longitudinal data are collected. However, such models are very useful from several points of view:

- substantively, many physiological parameters are controlled by endogenous biological oscillators, thus showing cyclical rhythmicity; similarly, human behaviour presents *behavioural regularity and continuity*, which is due to the inertia of each person's organism, tending to periodically repeat what has been done previously; summing up, past physiological conditions and behaviours explain future physiological conditions and behaviours,
- from a methodological point of view, the transversal models of data analysis (e.g., regression, mean comparisons …) assume the serial independence of the data (which implies no correlation), albeit in longitudinal studies, this principle of independence is transgressed since the longitudinal variable is very likely to be correlated with past values of itself,
- if the residuals are autocorrelated, and in temporary data it is the most frequent situation, then to make type I errors is very likely, in other words, to infer that there is an effect when there is not [33],
- if the values of the delayed variable are omitted as an IV, and this autoregressive variable is part of the explanatory model of the behaviour, the coefficients obtained are biased (i.e., they would not coincide with the true values of b_0_, b_1_,…) and they are inconsistent, this is, increasing the sample is not enough to obtain the correct coefficients of the model, which leads to incorrect confidence intervals for hypothesis testing, and the inferences drawn no longer have substantive meaning, [34, 35] and
- when analyzing temporary data, the autoregressive models are probably more appropriate (including autoregressive variables that are part of the process that generates the behavior) and parsimonious than the transverse ones (explaining the same temporary DV with fewer parameters).

The objective here is to compare the fit of a predominant previously used model (hr and hr^2^ as IVs, multilevel) to a multilevel autoregressive model. The design analysis is multilevel [31, 32], level 1 being each sAA record, which is then nested inside each participant, at level 2. Hence, the aim in this research is to determine which of the two models is better, statistically speaking, by examining the properties and the residuals of each model.

### Theoretical models

#### Hypothesis and model assumptions

We compare two competing explanatory models on sAA behaviour: the multilevel quadratic (the accepted model in previous literature) versus the multilevel autoregressive model (our proposed model). Our hypothesis is that sAA behaviour is multilevel at the intercept and is also a function of previous sAA behaviours; the two models are described and compared below.

#### Multilevel quadratic model

The traditional hypothesis is that sAA performance is generated as a function of the hr and the hr squared (hr^2^) of the record, or *sAA*_*j*,*t*_ *= f(hr*_*j*_, *hr*^*2*^_*j*_*)* [8, 36] its statistical representation being:

$${sAA}_{j,t}=b_{0j}+b_{1}\cdot {hr}_{j}+b_{2}\cdot {hr}_{j}^{2}+e_{j,t},$$ (1)

where *sAA*_*j*,*t*_ is the value of the variable sAA for participant *j* (in our case *j*: *1*, *2*, *…*, *19*) at one hr; *hr*_*j*_ is the hr of measurement (*9*, *10*, *11*, *…*, *21*) for participant *j*; the terms *b* represent the fixed regression coefficients (except the random coefficient *b*_*0j*_), and *e* is the set of errors of the equation.

In Eq 1 for *b*_*0j*_ we assume that the behaviour of the participants is multilevel (or random coefficient, i.e. the value of the random coefficient of intercept *b*_*0*_ in Eq 1 can be different for each participant in our sample). Eq 1 can then be transformed to represent the peculiarities of the specific level of each participant, where the fixed value of b_0_ is now transformed into level 2 coefficients:

$$Level\;2:\;b_{0j}=\gamma_{00}+u_{0j},$$ (2)

the properties of the elements in Eqs 1 and 2 being those commonly assumed in the literature [37–44].

Specifically, *b*_*0j*_ means that each individual may present particular characteristics with regards to each participant’s level coefficient in Eq 1. As a result, each participant can present their own deviation (*u*_*0j*_) of the intercept value, which is higher or lower than the general intercept of the sample (*γ*_*00*_), following a random normal distribution, and affecting the level of the variable sAA.

#### Multilevel autoregressive model

As an alternative hypothesis, we compare Eq 1 with an autoregressive (AR) model. In brief, an AR model of a variable presents a temporary dependence of previous values of the same variable, so in order to forecast the value of an AR variable, we only need to know the value of the previous record of that variable for any given subject. To describe the autoregressive model of salivary behaviour, if we suppose that a person’s sAA level at a certain time is a function of the amount of sAA shown by that same person in the previous *p* hr (*sAA*_*j*,*t*_ *= f(sAA*_*j*,*t-1*_, *sAA*_*j*,*t-2*_, *…*, *sAAs*_*j*,*t-p*_), then

$${sAA}_{j,t}=b_{0j}+b_{1}\cdot {sAA}_{j,t-1}+b_{2}\cdot {sAA}_{j,t-2}+\cdots +b_{k}\cdot {sAA}_{j,t-p}+e_{j,t},$$ (3)

where sAA is the value of the variable sAA for participant *j* at hr *t*; subscript *j* represents each participant (*j*: *1*, *2*, *…*, *19*); *t* is the moment of measurement, thus if we consider a value of sAA for a person *j* at a particular hr *t* (or *sAA*_*j*,*t*_), then the sAA value of this person *j* at the previous hr (*t-1)* will be *sAA*_*j*,*t-1*_, and so on until *sAA*_*j*,*t-p*_, *p* being the number of significant explanatory lags of sAA; the terms *b* and *e* are as in Eqs 1 and 2, though obviously they are different coefficients in the two equations because their statistical relationships are different; evidently, *b*_*0j*_ in Eq 3 is multilevel, its formulation being as in Eq 2 [15, 25, 28, 45–50].

#### Residual analysis

With respect to the residuals, *e*_*j*,*t*_, three empirical and theoretical assumptions have to be considered:

(1) The residuals fit a normal distribution. If the errors fit the condition of normality, then the parameters are unbiased, with minimum variance (efficient), and consistent (when the sample size increases, the estimators tend to the true population values). We use the Shapiro-Wilk statistical test to check for normality.

(2) They must be homoscedastic. If the residuals are not homoscedastic, then the coefficients (*b*_*0*_, *b*_*1*_,…) are not biased, but they do not have minimum variance, and the estimation of the *t*, *z*, *F* and *R*^*2*^ statistics are underestimated and not efficient [35]. To prove this assumption:

$$Var(e_{j,t})=\sigma_{2}\;(for\;all\;j\;and\;t)$$ (4)

Eq 4 is usually tested under two conditions: for participants, *Var(e*_*j*,*•*_*)*, and for hr, *Var(e*_*•*,*t*_*)*. Among the different statistical tests available, a robust test of Eq 4 can be performed by saving each value of *e*_*j*,*t*_ in Eqs 1 or 3, squaring their values ($e_{j,t}^{2}$), and estimating the regressions:

$$\begin{array}{l} e_{j,t}^{2}{=[1|h}_{T}]\cdot \left[\beta_{T}\right]{+a}_{j,t}\\ e_{j,t}^{2}={[1|h}_{J}]\cdot \left[\beta_{J}\right]+a_{j,t}^{\prime } \end{array}$$ (5)

where ***h***_***T***_ is a matrix of ‘dummy’ variables for the moments of measurement, of order (*J·(T-p))×(T-1*), and ***h***_***J***_ is a matrix of ‘dummy’ variables identifying the participants, with order *(J·(T-p))×(J-1)*. The *F* test of joint significance from the pooled regression is used, non-heteroscedasticity being accepted under the null hypothesis [27]. The results of Eq 5 are also very useful for detecting possible outliers on the values of the records in the sample.

(3) They must not present serial correlation (they should be ‘white noise’, without any statistically significant single autocorrelations and partial autocorrelations in the same lag order). If there is autocorrelation in the residuals, Kmenta (1971) [33] demonstrated that the parameters (*b*_*0*_, *b*_*1*_,*…*) are not biased, but the variances of the errors are underestimated. Therefore the variances and the standard errors of the parameters (that are in the denominator of the estimators) also tend to be underestimated and, likewise, the values of the *t*, *z*, *F* and *R*^*2*^ and *b*_*0*_, *b*_*1*_,*…* statistics are overestimated and not efficient, leading to type I errors, consisting of the assumption that a statistical effect exists, when in fact it does not.

Assumption (c), on serial correlation (obtained by previously saving the values of *e*_*j*,*t*_ in Eqs 1 or 3), can be tested in several different ways, one of them (under assumptions of normality and homoscedasticity) being the estimation of autocorrelations (*AC*_*1*_, *AC*_*2*_,…, *AC*_*k*_) and the partial autocorrelations (*PAC*_*1*_, *PAC*_*2*_,…, *PAC*_*k*_) of the residuals [51]. The value of *AC*_*k*_ is the correlation of the errors (*e*_*j*,*t*_) with previous *k* lagged values (*e*_*j*,*t-k*_):

$${AC}_{k}=r_{k}=Corr(e_{j,t},e_{j,t-k})=\frac{\sum (e_{j,t}\cdot e_{j,t-k})}{s_{e}^{2}},$$ (6)

in the previous numerator, we do not differentiate between *e*_*j*,*t*_ and *e*_*j*,*t-k*_ with respect to their corresponding means because the value of their means is zero. In the denominator, the standard error of *e*_*j*,*t*_ (*s*_*e*_) is the same as that of *e*_*j*,*t-k*_ (*s*_*e*_), because *e*_*j*,*t-k*_ is the same series of values as *e*_*j*,*t*_; its interpretation is the same as the traditional Pearson correlation. The *PAC* can be estimated by:

$${PAC}_{k}=\phi_{k}=Corr(e_{j,t},e_{j,t-k}\cdot e_{j,t-1},e_{j,t-2},\ldots e_{j,t-k-2},e_{j,t-k-1})=Corr[\{e_{j,t}–P(e_{j,t}|e_{j,t-1},e_{j,t-2},\ldots ,e_{j,t-k-1})\},\{(e_{j,t-k}–P(e_{j,t-k}|e_{j,t-1},e_{j,t-2},\ldots ,e_{j,t-k-1})\}]$$ (7)

P(W|Z) being the best linear projection of *W* on the vector of *Z* variables; a quicker procedure for the calculation of Eq 7 may be the Yule-Walker procedure [51]. The interpretation of *ϕ*_*k*_ is that its value is the correlation between *e*_*j*,*t*_ and *e*_*j*,*t-k*_ controlled by the rest of the variables that occupy an intermediate position between *e*_*j*,*t*_ and *e*_*j*,*t-k*_ (*e*_*j*,*t-1*_,*e*_*j*,*t-2*_,*…*, *e*_*j*,*t-k-1*_). The majority of the statistical programs have routines incorporated into them for computing the *AC* and the *PAC* values.

The researcher must check each of these three assumptions for the inner validation of the model, so if the residuals have the three properties mentioned above, all the participants would have the same statistical process.

#### Comparison of models

A model is a simplified representation of a certain reality and in statistics, where it entails a relationship between IVs and DV, a model can be interpreted as a simplified formalisation of reality through a data-generating process [52]. The properties of a good model include: (i) *parsimonious*, so that among two or more alternative models, the simplest must be selected, as it can explain the same with fewer variables or relationships among them; (ii) it has to have a significant *goodness of fit* (through *R*^*2*^, *F*, etc.); and (iii) it must be *unambiguous* in its predictions, comparing *prospective* predictions with the real results.

To compare the *goodness of fit* of two different models for the same data, when one of them is nested inside the other, we could use the statistical distribution of the overall fit (in the case of using multilevel regression models, the -*2loglikelhood*). When they are not nested models, which is our case, because the IVs are different in each model, we will use the Burnham and Anderson procedure [53, 54] based on the Akaike information criterion (AIC), which establishes that if Δ_i(AIC)_ = AIC_i_−AIC_min_, when Δ_i(AIC)_ > 7, then the model with the largest value is not supported, although Burnham and Anderson established more cut-off points. They also established an *evidence ratio* based on the relative likelihood of models [55]:

$$\frac{w_{min}}{w_{i}}=e^{\frac{1}{2}\cdot (\Delta_{i(AIC)})}$$ (8)

These concepts will now be applied to an empirical study, described below.

## Materials and methods

### Participants

Healthy Caucasian participants (9 male and 10 female) were recruited at Malaga University. To avoid possible confounds on the activity of SAA, smoking was considered an exclusion criterion in the present study. In a pre-study session, all participants underwent medical examination for past or current health problems and upon agreement to the study protocol they signed a written informed consent. Additional exclusion criteria were the presence of psychiatric, endocrine, cardiovascular, neurological or other chronic disease, medication with psychoactive drugs, beta-blockers or glucocorticoids, a body mass index (BMI) lower than 15 or higher than 28 kg/m^2^, and age below 20 or above 35 years. None of the participants reported significant dental or oral problems. Males had a *M* = 23.89 years of age *SD* = 3.51; females had a *M* = 22.50, *SD* = 1.51; there was not statistical differences in their means and in their variances. All the procedures carried out received ethical approval from the University of Malaga (CEUMA) and were performed in compliance with the Declaration of Helsinki [56].

We need to consider that the 19 participants are the level 2 of the sample, but the important data sample is the number of records of sAA (level 1) for each participant, thus our final number of data were 246 (18 participants with 13 records each and 1 participant with 12 records). Whilst obtaining more than 50 records is recommended in single level studies [51], it is important to note that our data are longitudinal and therefore quite different from cross-sectional data. Data and details of data organisation are shown in S1 Table and Tables A, B, C and D in S1 Appendix. A key aspect is that we can obtain the representativeness of the temporary process generator of the sAA data, more so than the representativeness of the participants. We did not calculate the a priori size of the sample, because there is no previous evidence of the use of AR models in sAA.

### Laboratory analysis and procedure

For saliva sampling, the passive drooling method was modified from the original procedure suggested by Navazesh [57] as described in Rohleder et al. [58]. Participants were instructed to first void their mouths of saliva by swallowing. By contrast to the original method, saliva was allowed to accumulate passively for 2 min. Participants then spit all saliva into 10-ml polystyrene tubes. Saliva samples were stored at -20b0C until analysis. After thawing, tubes used for passive drooling were centrifuged at 20,000 rpm for 5 min, resulting in mucous compounds being restricted to the lower part of the tube. Saliva was then diluted 1:750 in distilled water. sAA activity was determined using a chemistry analyser and an amylase assay kit (Dimension®, DADE Behring, USA). Saliva flow rate was calculated by the gravimetric method but not analysed for the purpose of this study. The first sample was collected at 09:00 hr and the same procedure was carried out every hr by the same laboratory personnel until 21:00 hr. Therefore, a total of 13 measurements were made for each participant. The participants were in laboratory during the 12 hr with ‘constant-routine protocol’: phones or any electronic devices were not allowed, ‘piped music’ was played in the background, and participants had travel magazines for reading, and disposed of a chair, a table, and a reclining stretcher; this protocol is designed to examine human biorhythms without interference of an environmental or behavioural nature [59]. The laboratory environment was controlled (24–25°C, light intensity of < 120 lx).

With the aim of normalising the data, each resulting value was then transformed into the Napierian logarithm of the enzymatic activity of sAA. The data are shown in S1 Table, which also indicates how the mean sAA activity follows an upward trend with a stable variance. A total of 246 records were collected (13 for each of the 19 participants). However, at 21:00 hr participant 15 presented an outlier value, which was omitted from the analyses. Data analyses were performed with the SPSS [60] and Stata [61] statistical programs. The descriptive analysis was conducted with SPSS and the model analysis with both programs.

### Results

The inter-participant and inter-hour variability are shown on S1 Table, the inter-participant by hr is on the last raw (*s*_•,*t*_), and the inter-hour by participant in last column (*s*_*j*,•_). The Levene test of homoscedasticity shows significant variability inter-participants sAA values, but not significant variability inter-hour values.

Two types of data analysis were performed: (i) sAA as a function of *hr* and *hr2*, and (ii) sAA as an autoregressive model. The statistical models have been estimated using maximum likelihood in SPSS and Stata, because SPSS and Stata have different ways of entering data.

#### Data analysis 1 (*M1*): *sAA* as a function of *hr* and *hr*^*2*^

The data in S1 Table are organised using a ‘person-level dataset’ system (or ‘one participant per row’ [14]. However, for a multilevel autoregressive analysis we have changed the configuration to a ‘person-period dataset’ system (so that each person provides multiple records, one for each time of measurement). Thus, our file will have, at first, *J·T* data, or 19·13 = 247 lines of data. Note, however, that the last value of participant 15 is missing, thus we have 246 values. Paragraphs (i) and (ii) of S1 Appendix show an easy example of the organisation of data in SPSS, from the ‘person-level dataset’ to the ‘person-period dataset’ system [14], so as to be able to analyse them by means of the function: *sAA*_*j*,*t*_ *= f(hr*_*j*_, *hr*^*2*^_*j*_*)*.

The intraclass correlation (ICC) yields a value of .702 [(*σ*_*uo*_*)*^*2*^*/{*(*σ*_*uo*_*)*^*2*^*+*(*σ*_*e*_*)*^*2*^*} =* 0.887/(0.887+0.377)]. This indicates that 70.2% of the variability of the results in sAA (level 1) within the same participant (level 2) resembles the variability of sAA in other participants. Thus, the hypothesis of the multilevel model does seem feasible. The value of -2LL is 523.735 and that of AIC is 529.735.

We have developed the estimation regression Eq 3, and the results can be seen in S2A–S2C Table.

The statistical formulation of *sAA*_*j*,*t*_ *= f(hr*, *hr*^*2*^*)* is:

$${sAA}_{j,t}=(0.157+u_{0j})+0.400\cdot {hr}_{j,t}-0.010\cdot {hr}_{j,t}^{2}+e_{j,t,}$$ (9)

**Fig 1:**
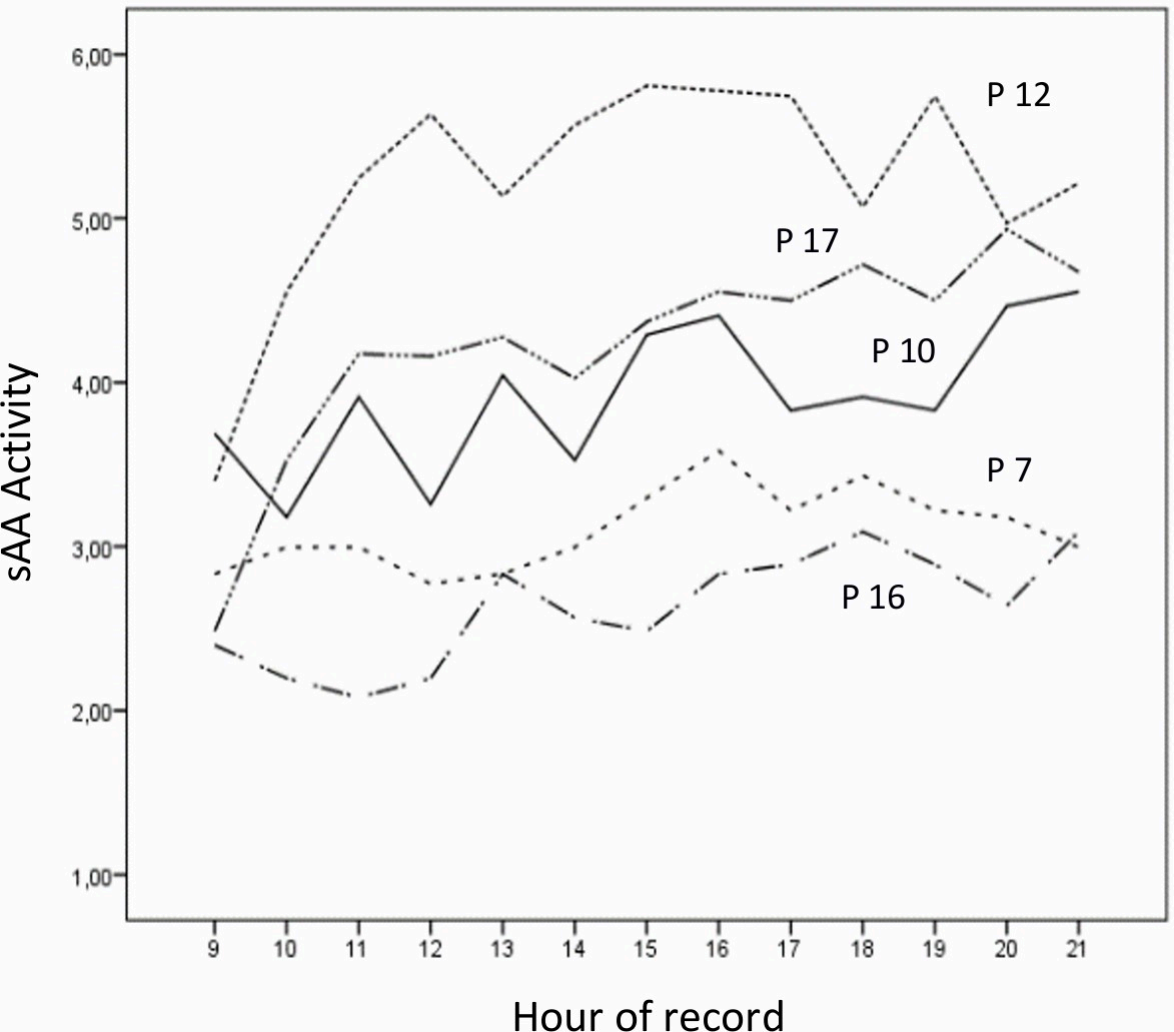
Original sAA records of participants (P) 7, 10, 12, 16 and 17.

**Fig 2:**
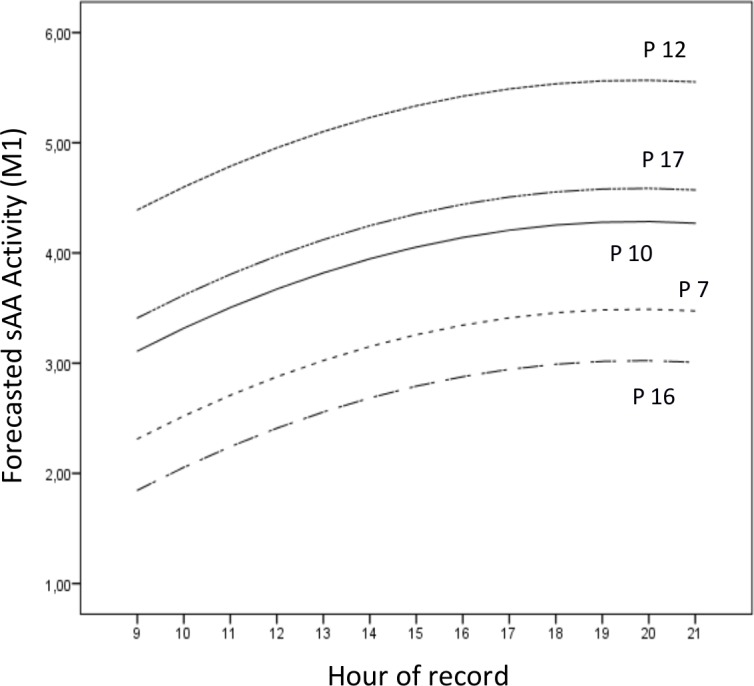
Forecasted values of P7, P10, P12, P16 and P17 (the same participants as in Fig 1), in accordance with Eq 9 (*M1*), from 09:00 to 21:00 hr.

In Fig 1 we show the real sAA values for participants 7, 10, 12, 16 and 17. We do not represent all the participants because the figure would contain many lines that cross each other, thus making it difficult to read. In Fig 2 we show the forecasted values of sAA according to Eq 9 for the same participants for the interval from 9:00 to 21:00 hr.

On analysing the residuals of Eq 9, with regard to assumption (1) on the normality of the residuals, we obtain a Shapiro-Wilk test value of .*996(246 df)*, *p =* .*858*, so we do not reject the normality of the residuals.

We have calculated the values of each *e*_*j*,*t*_ squared in Eq 9, to test assumption (2), and on estimating its parameters in Eq 5, with the hr dummies we obtain *F(12*, *233) = 1*.*083*, *p =* .*375*, and with the participant dummies, *F(18*, *227) = 1*.*561*, *p =* .*072*, without rejecting the homoscedasticity of the residuals for hr and participants.

We have calculated the ACs and the PACs of the residuals in Eq 9, to test assumption (3), the first two being statistically non-significant, but the third residuals being significant (*AC*_*3*_ = *-*.*174*, *n = 189*, *p =* .*017*, and *PAC*_*3*_ = .*184*, *n = 185*, *p =* .*012*), so technically these residuals are not ‘white noise’, and the values of the statistical tests of the parameters in Eq 9 are overestimated, and not efficient, leading to type I errors.

On comparing -2LL of *M*, 399.226, and the null model, 523.735 (the null model has only the intercept and is nested inside *M1*, the null model is used only to test hypotheses on statistical validity of the compared model, with more parameters), Δ_(-2LL)_ = 523.735–399.226 = 124.509, 2 d.f., p < .001, so *M1* is statistically better than the null model.

#### Data Analysis 2 (*M2*): *sAA* as an autoregressive model

The ICC is the same as in *M1*. For the estimation of the correct number of *p* lags in an *AR(p)* model [51], we have calculated the *ACs* of *sAA*_*j*,*t*_: *Corr(sAA*_*j*,*t*_, *sAA*_*j*,*t-1*_*)*, *Corr(sAA*_*j*,*t*_, *sAA*_*j*,*t-2*_*)*, *…*, *Corr(sAA*_*j*,*t*_, *sAA*_*j*,*t-k*_*)* and its *PACs*: *PAC(sAA*_*j*,*t*_ *sAA*_*j*,*t-1*_*)*, *PAC(sAA*_*j*,*t*_, *sAA*_*j*,*t-2*_*·sAA*_*j*,*t-1*_*)*, *…*, *PAC(sAA*_*j*,*t*_, *sAA*_*j*,*t-k*_*·sAA*_*j*,*t-1*_ *sAA*_*j*,*t-2*_,*…*, *sAA*_*j*,*t-k-1*_*)*, observing that the values of the correlations decrease slowly and *PAC* is not significant after the third lag. Hence, for Eq 3 a second-order autoregressive (*AR(2)*) model is proposed (*sAA*_*j*,*t*_ *= f(sAA*_*j*,*t-1*_, *sAA*_*j*,*t-2*_):

$$\begin{array}{l} sAA_{j,t}=b_{0j}+b_{1}\cdot sAA_{j,t-1}+b_{2}\cdot sAA_{j,t-2}+e_{j,t}\\ sAA_{j,t}=0.562+0.523\cdot sAA_{j,t-1}+0.365\cdot sAA_{j,t-2}+e_{j,t} \end{array}$$ (10)

In Eq 10 we find that the random multilevel coefficient *u*_*0j*_ is not statistically significant, so we take out this coefficient, thus obtaining the results in S4A–S4C Table.

**Fig 3:**
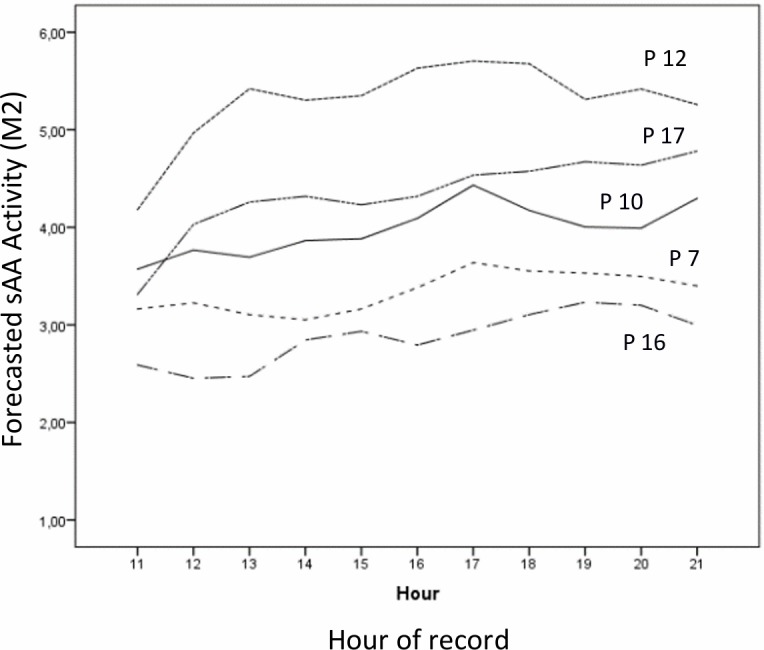
Forecasted sAA values of P7, P10, P12, P16 and P17, in agreement with Eq 10 (*M2*), from 11:00 to 21:00 hr.

In Fig 3 we have represented the forecasted values according to Eq 10 at each hr. Note that there are only forecasted values from 11:00 hr, since we have missed the first two values of each participant. In time series, each AR lag generates a missing data of 1 value per participant. Since we have two lags, i.e. AR(2) model, we miss 2 values per participant (38 values in total, 19*2) [27, 51]; this implies that for the final autoregressive analysis we have a total of 208 values (246–38). We have not made any estimation of missing values because the standard estimation systems are planned for transversal data, not for autoregressive data. In an example in S1 Appendix, paragraph (iii), there is an easy example to perform AR(2) analysis with 3 participants and 5 records each, organising data with an ‘person-period dataset’ system.

By saving the residuals in Eq 10, and on analysing the normality of the residuals with the Shapiro-Wilk statistical test, its value is .*992 (208 df)*, *p =* .*325*, so the residuals fit a normal distribution, proving assumption (1) on residuals.

On testing for the non-heteroscedasticity of the errors in Eq 10 (by applying Eq 5), with the hr dummies we obtain: *F(10*, *197) = 1*.*383*, *p =* .*190* and for participants: *F(18*, *189) = 1*.*541*, *p =* .*080*. Thus, we can accept the non-heteroscedasticity, assumption (2) of the data according to the hr of measurement and for the participants.

We have estimated the ACs and the PACs of the residuals in Eq 10, to test assumption (3), all of them being statistically non-significant, and so technically these residuals are ‘white noise’.

Comparing *M2* with the null model, Δ_(-2LL)_ = 523.735–316.000 = 207.735, 2 d.f., p < .001, so the overall statistical fit is significant, fitting *M2* to data. If we compare *M1* with *M2*, we observe that they are not nested models, so it is necessary to compare their AIC, Δ_i(AIC)_ = 409.226–316.000 = 93.226 (a lot higher than the value of 7), and the *evidence ratio* between the two models based on Eq 8 is 1.75·10^20^, meaning that *M2* is more likely than *M1* to be a good statistical representation of our data. Another evidence that *M2* is better than *M1* is that the total variance of the errors of *M2* is .257 ($s_{e}^{2}$, S3C Table), whereas the total variance of the errors in *M1* is 1.115 ($s_{e}^{2}+s_{u}^{2}$, S2C Table), this is, the total of the residual errors of *M1* is four times bigger than for *M2*.

**Fig 4:**
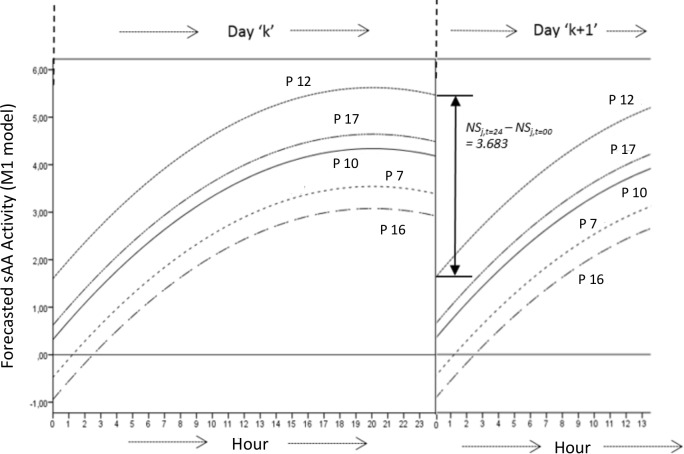
Forecasted values of P7, P10, P12, P16 and P17, in agreement with Eq 9 (*M1*) for a complete day (from 00:00 to 24:00 hr), and part of the next day (from 00:00 to 13:00 hr). Note the gap of 3.683 units of forecasted values for sAA when hr = 24:00 or hr = 00:00.

Additionally, *M2* has better predictive power than *M1*, so in Eq 9, if we forecast the expected value for *t = 24*:*00* hr, and for ‘the fixed intercept’ value (*b*_*0*_ *= γ*_*00*_ = .*157*), we will obtain: $\hat{{NS}_{J,24=(.157+0)+.400\cdot 24-.010\cdot 242=3.840}}$. However, a moment later, if we take *t = 00*:*00* hr (observe that 24:00 hr = 00:00 hr), the forecast would be: $\hat{{NS}_{J,0}}=(.157+0)+.400\cdot 0-.010\cdot 02=.157$, which means that *M1* yields two different forecasted values for the same moment: *3*.*840* and .*157*, with a difference of *3*.*683* points of sAA. By applying a t Student-Fisher test for this difference of values, however: *t = 8*.*327 (gl = 18*, *p <* .*001)*, which means that for all the participants and for the same time there are two different forecasted values, with statistical differences. Furthermore, there are seven participants (*3*, *6*, *7*, *9*, *11*, *15* and *16*) with sAA negative values, which is not correct. We can see this with participants *7*, *10*, *12*, *16* and *17* in Fig 4, where the difference for the same person from hr = *24*:*00* to hr = *00*:*00* is *3*.*683* in sAA values, and participants *7* and *16* have negative forecasted sAA values at 00:00 hr.

A model must have a good *prospective* projection, so it must have a ‘long-run equilibrium point’[35]. In a dynamic model, as for example, in Eq 10, there is a long-run equilibrium point when *sAA*_*j*,*t*_ *= sAA*_*j*,*t-1*_ *= sAA*_*j*,*t-2*_ = sAA^*^, so *e*_*j*,*t*_ = 0, and then, substituting these values in Eq 10, we have:

$${sAA}^{*}=.562+.523\cdot {sAA}^{*}+.365\cdot {sAA}^{*}$$ (11)

and on isolating sAA^*^, we obtain sAA^*^ *= 5*.*018*. This means that the ‘equilibrium point’ of the sAA time series has a value of *5*.*018*, this being the general tendency of the series. Conversely, if we want to do a long run time forecasting of *M1*, it would have to be: hr → ∞, so in Eq 9: *sAA*_*j*,∞_ *= 0*.*157 + 0*.*400·*∞ - *0*.*010·*∞^*2*^ = —∞, we can see this negative trend on Fig 4.

#### Data Analysis 3 (*M3*): sAA as function of *hr*_*j*_, *hr*^*2*^_*j*_, and *sAA*_j,t-1,_ *sAA*_*j*,*t-2*_

As an additional check, we have introduced within the same model the four IVs used in *M1* and *M2*: random intercept (*b*_*0j*_), *hr*_*j*_, *hr*^*2*^_*j*_, and *sAA*_*j*,*t-1*,_ *sAA*_*j*,*t-2*_, obtaining the results on S4A–S4C Table, where it is shown (S4B Table) that only the coefficients of *sAA*_*j*,*t-1*_ and *sAA*_*j*,*t-2*_ are significant, and its values are very similar to those in Eq 10; conversely, the coefficients of *hr* and *hr*^*2*^ change, even of sign, with respect to those of *M1*, being unstable and not significant. The S4 Table results are additional clues that *M2* is better than *M1*.

Therefore, we show that for sAA, autoregressive *M2* (*sAA*_*j*,*t*_ *= f(sAA*_*j*,*t-1*_, *sAA*_*j*,*t-2*_*)*) is a better model than *M1* (*sAA*_*j*,*t*_ *= f(hr*, *hr*^*2*^), and we can accept Eq 10 as a better system than Eq 9 for the representation of sAA data generation.

## Discussion

The *AR(2)* equation of *M2* indicates that to be able to predict the sAA value for any moment and for any given participant (*sAA*_*j*,*t*_) it is only necessary to determine the value of the variable sAA for that participant (‘*j*’) just one hr immediately before (*sAA*_*j*,*t-1*_) and two hr before (*sAA*_*j*,*t-2*_), as in Eq 10, so our hypothesis is supported with respect the AR model (2), but not in regard to the multilevel intercept. In other words, in functional terms, *sAA*_*j*,*t*_ *= f(sAA*_*j*,*t-1*_, *sAA*_*j*,*t-2*_*)*, meaning that the series ‘memorise’ the two previous values corresponding to one hr and two hr before the recorded moment. Chiefly, sAA activity is regulated by the autonomic nervous system and depends only secondarily on the external time of day, and even though the series has a growing or a decreasing tendency, that tendency is memorised and autocontrolled by the very inertia of the series according to the previous two sAA values obtained hourly.

The results achieved in the random ANOVA for calculating the ICC of sAA yielded a difference of means among the participants that was significant (and with a very high ICC), which might suggest that the resulting model (*M2*) would also be multilevel, at least in the constant, *b*_*0*_. Yet, it was not, probably because in *M2* the previous values of *sAA*_*j*,*t*_ (*sAA*_*j*,*t-1*_, *sAA*_*j*,*t-2*_) also ‘memorise’ the level of the time series, so when a person has high values in the *sAA*_*j*,*t-1*_ or *sAA*_*j*,*t-2*_ measures, he or she will also have a high value in *sAA*_*j*,*t*_ (and vice-versa). It is therefore no longer necessary, statistically speaking, to include the random intercept, *u*_*0j*_, which represents the deviation of each participant with respect to the fixed intercept.

The variables hr and hr^2^ were statistically significant in this research (*M1*), although when we compare the *AIC* results in *M1* and *M2*, we see that the *AIC* values in *M2* are better than in *M1*, and thus *M2* is accepted as an improved model for our data. We have also performed a regression analysis with *sAA*_*j*,*t-1*_, *sAA*_*j*,*t-2*_, hr and hr^2^ (*sAA*_*j*,*t*_ *= f(sAA*_*j*,*t-1*_, *sAA*_*j*,*t-2*_, *hr*, *hr*^*2*^*)*), only the coefficients of *sAA*_*j*,*t-1*_, *sAA*_*j*,*t-2*_ being statistically significant (and their values are very similar to the values in Eq 10, in other words, *sAA*_*j*,*t-1*_ and *sAA*_*j*,*t-2*_ have very stable coefficients), which provides additional evidence that the *AR(2)* model is better than the *hr* and *hr*^*2*^ model.

Hence, the *AR(2)* model (*sAA*_*j*,*t*_ *= f(sAA*_*j*,*t-1*_, *sAA*_*j*,*t-2*_*)* is a more suitable model than *M1* (*sAA*_*j*,*t*_ *= f(hr*, *hr*^*2*^*)* given that:

- It fits better, because the variance of the *M2* residuals is considerably smaller than that in *M1*.
- The *AIC* value of *M2* is much smaller than that of *M1*, and the *evidence ratio* is also very high in favour of *M2*.
- *M2* is statistically more parsimonious, because it has four free parameters (*b*_*0*_, *b*_*1*_, *b*_*2*_ and in Eq 10), while *M1* has five free parameters (*b*_*0*_, *b*_*1*_, *b*_*2*_, $s_{e}^{2}$ and $s_{u}^{2}$ in Eq 9).
- *M2* has better predictive power than *M1*; for the long run equilibrium point, the expected value of *sAA*_*j*,*t*_ in the *AR(2)* model is *5*.*018*, but in the *M1* model its value is meaningless (-∞) and, similarly, the forecasted values in *M1* for 00:00 and 24:00 hr are ambiguous, with two different results.
- The time of day may be a ‘memory’ function that can be also influenced by the same *sAA* value of the previous day, i.e. *sAA*_*j*,*t*_ could also be influenced by *sAA*_*j*,*t-24*_, which is the amount of *sAA* twenty-four hr earlier (*sAA*_*j*,*t*_ = *f(sAA*_*j*,*t-1*,_ *sAA*_*j*,*t-2*_,*…*, *sAA*_*j*,*t-24*_*)*). Hence, if we had taken values over more than one day, the model could have been:

$${sAA}_{j,t}=b_{0}+b_{1}\cdot {sAA}_{j,t-1}+b_{2}\cdot {sAA}_{j,t-2}+b_{3}\cdot {sAA}_{j,t-24}+e_{j,t}$$ (12)

This would indicate that the sAA value at any hr (*sAA*_*j*,*t*_) could be forecasted by establishing the sAA value one hr before (*sAA*_*j*,*t-1*_), the sAA value two hr before (*sAA*_*j*,*t-2*_) and the sAA value twenty-four hr before (*sAA*_*j*,*t-24*_). Note that the sAA value twenty-four hr before is the sAA value of the same hr on the previous day (*sAA*_*j*,*t*_ *= f(sAA*_*j*,*t-1*_, *sAA*_*j*,*t-2*_, *sAA*_*j*,*t-24*_*)*). In brief, *sAA* very likely presents a daily cyclic pattern of approximately 24 hr, taking into account that hr is the unit of measurement in this investigation. In our hands, sAA behaviour fit a circadian pattern, with the lowest values being observed during the morning hours, followed by a steady increase that extended well into the evening. This is only partially in agreement with previous results. Previous research has shown that sAA, like cortisol, exhibits a diurnal activity, with concentrations declining from 60 min after waking and increasing gradually thereafter with peak in the afternoon [8]. We acknowledge that our conclusions are limited to diurnal variations and therefore future research should be expanded to include nocturnal periods of activity. In addition, it would be convenient to obtain data of the same participants in two consecutive days with 24 hr of difference, i.e.: two consecutive days from 9 to 14 hr, another participant from 10 to 15 hr two days in a row, and so on… for the purpose of estimating the circadian AR dependency: value of *sAA*_*j*,t_ 24 hr before, *sAA*_*j*,*t-24*_.

It must be noted that, in the *AR(2)* analysis, the first two data records (9:00 and 10:00 hr) are missing for each participant [14, 40], which means that the final regression in *M2* is performed over 208 records (19 participants forecasting 11 hr, except 21:00 hr for participant 15). In contrast, in *M1*, only the record for 21:00 hr for participant 15 was missing, the analysis thus being performed over 246 records (19 participants forecasting 13 hr, except 21:00 hr for participant 15). Furthermore, despite the fact that the estimation of the regression parameters becomes less powerful as the number of data decrease, in *M1* one more significant parameter was obtained than in *M2* (the random intercept is lost in *M2*).

On observing the data obtained directly from each participant and the mean for each respective hr shown in S1 Table, it is evident that they follow a growth tendency in the time interval that was measured (from 9:00 to 21:00 hr). An analyst would have probably recommended differentiating the series before beginning the autoregressive analyses. Such a differentiation is, however, not altogether empirically, analytically or theoretically correct, as will be detailed in the following points:

- If it is assumed that the series has to be differentiated, then this is equivalent to saying that the variable *sAA* does not have a stable mean; yet, in a normal person, any psychobiological variable can be expected to move within certain maximum and minimum values (from the tendency of the data alone, it is most unlikely that *sAA* will increase indefinitely or decrease below a *sAA* value of zero), which makes it unnecessary to differentiate the series. In other words, a sample of *sAA* values (with a slowly ascending trajectory) was taken from data that, in the long term, are stationary because if we took samples over several consecutive days at one-hr intervals, the sample would become stabilised, rising during the day and down during the night, and returning each day to its previous values.
- On analysing the data correctly, it is found that all the parameters in *M2* are significant, with positive values that are ‘persistent’, stable in the long run [51]. Moreover, the residuals are seen to be ‘white noise’ without heteroscedasticity, which is further evidence that it is not necessary to differentiate the series.
- According to Engle and Granger’s [62] cointegration model, if a series is differentiated, the model will only be valid for that interval, but it is not valid for a long-term prediction. In other words, if the series is not differentiated, the model thus obtained describes the whole series (and future values) well, regardless of the tendency of the sample interval. The model is therefore valid whether the series has a growing, descending or horizontal sample tendency.

One important point to be highlighted is that neither the variable hr nor the hr^2^ have been included as IVs in the best final model, *M2*. This omission is due to two reasons: (i) because they are not significant, and (ii) in order to focus on the autoregressive aspects of the data. However, it is not wise, in general, to fit the variable sAA (or any other psychological, physiological or biological variable for the same matter) according to the hr or powers of the hr (*hr*^*2*^, *hr*^*3*^, etc.) because if we had extended the measurement interval of the hr, then the series would have changed tendency outside the sample interval that was measured (and this is what is most likely to happen), the shape of the function of the series would also change, and hence new (cubic, fourth degree or higher degree: *hr*^*3*^, *hr*^*4*^, etc.) parameters would have to be included. Thus, the variable sAA has a minimum mean value at 9:00 hr and a maximum at 21:00 hr, and it is more than likely that as of a certain time (probably after 21:00 hr) it starts to descend until a minimum level is reached at around 7:00 to 09:00 hr the following day.

In short, *M2* (*sAA*_*j*,*t*_ *= f(sAA*_*j*,*t-1*_, *sAA*_*j*,*t-2*_*)*) is statistically and conceptually better than *M1* (*sAA*_*j*,*t*_ *= f(hr*, *hr*^*2*^*)*) because (i) the errors in *M2* are ‘white noise’ but those in *M1* are not; (ii) the errors (the variance and the AIC) are smaller in *M2* than in *M1;* (iii) *M2* has fewer parameters than *M1;* (iv) the forecasted values of *M2* are better: no negatives, inside the real limits of *sAA*_*j*,*t*_, and unambiguous. Thus, sAA has its own (autoregressive) inner memory, depending on two previous values, rather than depending on the hr of the day.

As a final orientation, in our opinion (i) methodologists in life and social sciences should receive more training in analysing ILM, both on statistical modelling and on model validation through the corresponding residual analysis; (ii) there should be more collaboration between researchers who are experts in substantive areas and methodological analysis, with the aim of producing more appropriate statistical models that explain the process of generating the data; (iii) as an additional resource for researchers’ and methodologists’ training and comparison of analytical methods, scientific journals ought to ask the researchers/authors who have used original datasets for a copy of the data (as a table in the paper or in digitised format) and upload and publish them on the internet with free access for the entire scientific community; and (iv) when the data analyst knows how to organise the longitudinal data in each statistical program, it is not necessary to use an specialised program in panel data time series in order to analyse ILM, because the results are the same.

The increase in ILM research (which is longitudinal and measures different variables) conducted in different participants using telemetry or other techniques [63, 64], together with the use of methods of analysis that are better suited to this kind of data are expected to represent a step forward in scientific understanding, in the prevention of biological or psychological disorders and in improving the quality of human life.

## Supporting information

S1 Appendix Different ways of organising the data. (DOCX)

S1 Table sAA for each participant, according to the time. **The means and standard deviations are added for each participant (*sAA*_*j*,■_ and *s*_*j*,■_) and for each time (*sAA*_■,*t*_ and *s*_■,*t*_).** ^a^ Values of 0 mean that the original value of the salivary variable is 1 (ln(1) = 0).
(DOCX)

S2 Table Results of the multilevel regression of *M1*. *DV*: *sAA*_*j*,*t*_, *IVs*: *hr*, *hr*^*2*^.
(DOCX)

S3 Table Results of the multilevel regression of *M2*. DV: sAA_j.t_, IVs: sAA_j,t-1_, sAA_j,t-2_.
(DOCX)

S4 Table Results of *M3*, multilevel regression with *DV*: *sAA*_*j*.*t*_, *IVs*: *hr*, *hr*^*2*^, *sAA*_*j*,*t-1*_, *sAA*_*j*,*t-2*_. (DOCX)
