# Correction: Intensive longitudinal modelling predicts diurnal activity of salivary alpha-amylase

**DOI:** 10.1371/journal.pone.0225745

**Published:** 2019-11-19

**Authors:** Jesús F. Rosel, Enrique F. Maldonado, Pilar Jara, Francisco H. Machancoses, Jacinto Pallarés, Pedro Torrente, Sara Puchol, Juan J. Canales

Enrique F. Maldonado should be listed as the second author, and his affiliation is 1: Clinical Neuropsychology Laboratory, School of Psychology, University of Malaga, Andalucia Tech, Malaga, Spain. The contributions of this author are as follows: Conceptualization, data curation, investigation, methodology.

The correct citation is: Rosel JF, Maldonado EF, Jara P, Machancoses FH, Pallarés J, Torrente P, et al. (2019) Intensive longitudinal modelling predicts diurnal activity of salivary alpha-amylase. PLoS ONE 14(1): e0209475. https://doi.org/10.1371/journal.pone.0209475
