# Supplementary material for: Intensive longitudinal modelling predicts diurnal activity of salivary alpha-amylase
Source: PLoS One. 2019 Jan 23;14(1):e0209475. doi: 10.1371/journal.pone.0209475 (PMC6343885; doi:10.1371/journal.pone.0209475)
Supplement: S1 Appendix — (DOCX) [file pone.0209475.s001.docx]

**Appendix**

**Different ways of organising the data**

We present the different forms of data organisation of a data file, from ‘one participant per row’ data to a ‘person-period dataset’ for a *sAA_j,t_ = f(hr, hr^2^)* function and to a ‘person-period dataset’ for an *AR(p)* function. However, each data analyst should organise the data according to the statistical program used.

**(i) ‘One participant per row’ data**

To organise data in a PTS model, we will describe one easy example between a quadratic model and an autoregressive model. Thus, the general system for organising data for analyses in a *quadratic model* would be, for three people *(j=3)* and five moments *(t=5, hr = 09:00, 10:00,…,13:00)*, to start from ‘one participant per row' data (Singer & Willett, 2003), as data organised in Table A.

**Table A. Example of ‘one participant per row' data**

**(ii) ‘Person-period dataset’ for an *sAA_j,t_ = f(hr, hr^2^)* function**

For a multilevel autoregressive analysis, we change the configuration to a ‘person-period dataset’, so if ***Y = X·β + e,*** with ***X = [ 1 | h | h^2^ ]***, as in Table B.

**Table B. Example of ‘person-period dataset’ data without autoregressive variables in form of equation**

**(iii) ‘Person-period dataset’ for an *AR(p)* function**

In an *AR(p)* model we must reorganise the data if we have an *AR(p)* model, placing as many line gaps as there are lags in the model or even more, to ensure a correct analysis (*p* or more gaps); hence, if we had an *AR(2)* model, in our case *sAA_j,t_* = f(*sAA_j,t-1_*, *sAA_j,t-2_*), for three people *(j=3)* and five moments *(t=5, hr = 9, 10,…,13 )*:

***Y = X·β + e,*** with ***X = [ 1 | sAA_j,t-1_ | sAA_j,t-2_ ]***. In SPSS, or in any other omnibus statistical program, it is necessary to introduce the data as in Table C.

**Table C. Example of ‘person-period dataset’ data with autoregressive variables *sAA_j,t-1_* and *sAA_j,t-2_* expressed as an equation**

Therefore, in an *AR(p)* model, we miss the first *p* values of each participant (*j*), and if there are *t* moments measured for each participant, we would now have *j·(t-p)* complete values. In our example, we have: *p = 2, j = 3*, and *t = 5,* so we omit: *p·j = 2·3 = 6* values, and the valid number of values is: *j·(t-p) = 3·(5-2) = 9*. These 9 complete values for analysis are highlighted with green shading in C Table. We miss the first two values of each participant [14, 40].

Observe that:

(a) there is no problem in analysing data in C Table with an omnibus software statistical program because, by default, the majority of statistical programs use ‘LISTWISE’ for analysis, this is, they use the complete lines of variables used in the requested analysis, so they will only use, for each participant, *sAA_j,3_*, *sAA_j,4_* and *sAA_j,5_* as values of DVs ;

(b) if we had not made the *p* gaps between participants, we would have made (erroneously) the first values of each participant a function of the last values of the previous participant in the data matrix, e.g.: *sAA_2,1_* = f(*sAA_1,5_*, *sAA_1,4_*), *sAA_2,2_* = f(*sAA_2,1_, sAA_1,5_*), and *sAA_3,1_* = f(*sAA_2,5_*, *sAA_2,4_*), *sAA_3,2_* = f(*sAA_3,1_, sAA_2,5_*), so the results would have been spurious.

**(iv) Introduction of data in Stata**

The same example of 3 participants with 5 records each, in Stata data input, Panel Data module, would be the same matrix of A Table, but transposed (Table D).

**Table D. How to enter the data for analysis in Stata, Panel Data module**

So, for each participant, the temporary data must be written in columns. The system of introduction of data is easier than in SPSS, but Stata reorganises the data internally as in C Table of this Appendix, so if the experimenter is clear about the organisation of data for autoregressive analysis, there is no need for any special panel data analysis program. We have performed the regression corresponding to Equation 10, organised the data as in C Table, with the Linear Mixed Models option in SPSS, and with the Longitudinal/Panel Data in Stata software, organising data as in SD Table, obtaining the same results.

**SA Table.**

**Table A. Example of ‘one participant per row' data**

|  | Hour | | | | |  |
| --- | --- | --- | --- | --- | --- | --- |
|  | 09:00 | 10:00 | 11:00 | 12:00 | 13:00 |  |
| Participant 1 | *sAA_1,1_* | *sAA_1,2_* | *sAA_1,3_* | *sAA_1,4_* | *sAA_1,5_* |  |
| Participant 2 | *sAA_2,1_* | *sAA_2,2_* | *sAA_2,3_* | *sAA_2,4_* | *sAA_2,5_* |  |
| Participant 3 | *sAA_3,1_* | *sAA_3,2_* | *sAA_3,3_* | *sAA_3,4_* | *sAA_3,5_* |  |

**SB Table.**

**Table B. Example of ‘person-period dataset’ data without autoregressive variables in form of equation**

| ***Y*** | *=* | ***1*** | ***h*** | ***h^2^*** | *** | ***β*** | *+* | ***e*** |  |
| --- | --- | --- | --- | --- | --- | --- | --- | --- | --- |
| *sAA_1,1_* |  | 1 | *9* | *81* |  |  |  | *e_1,1_* |  |
| *sAA_1,2_* |  | 1 | *10* | *100* |  |  |  | *e_1,2_* |  |
| *sAA_1,3_* |  | 1 | *11* | *121* |  |  |  | *e_1,3_* |  |
| *sAA_1,4_* |  | 1 | *12* | *144* |  |  |  | *e_1,4_* |  |
| *sAA_1,5_* |  | 1 | *13* | *169* |  |  |  | *e_1,5_* |  |
| *sAA_2,1_* |  | 1 | *9* | *81* |  |  |  | *e_2,1_* |  |
| *sAA_2,2_* |  | 1 | *10* | *100* |  | *b_0_* |  | *e_2,2_* |  |
| *sAA_2,3_* | = | 1 | *11* | *121* | * | *b_1_* | + | *e_2,3_* |  |
| *sAA_2,4_* |  | 1 | *12* | *144* |  | *b_2_* |  | *e_2,4_* |  |
| *sAA_2,5_* |  | 1 | *13* | *169* |  |  |  | *e_2,5_* |  |
| *sAA_3,1_* |  | 1 | *9* | *81* |  |  |  | *e_3,1_* |  |
| *sAA_3,2_* |  | 1 | *10* | *100* |  |  |  | *e_3,2_* |  |
| *sAA_3,3_* |  | 1 | *11* | *121* |  |  |  | *e_3,3_* |  |
| *sAA_3,4_* |  | 1 | *12* | *144* |  |  |  | *e_3,4_* |  |
| *sAA_3,5_* |  | 1 | *13* | *169* |  |  |  | *e_3,5_* |  |

**SC Table.**

**Table C. Example of ‘person-period dataset’ data with autoregressive variables *sAA_j,t-1_* and *sAA_j,t-2_* expressed as an equation**

| ***Y*** | *=* | ***1*** | ***sAA_j,t-1_*** | ***sAA_j,t-2_*** | ***h*** | ***h^2^*** | *** | ***β*** | | *+* | ***e*** |  |
| --- | --- | --- | --- | --- | --- | --- | --- | --- | --- | --- | --- | --- |
| *sAA_1,1_* |  | 1 | - | *-* | *9* | *81* |  |  | |  | *e_1,1_* |  |
| *sAA_1,2_* |  | 1 | *sAA_1,1_* | *-* | *10* | *100* |  |  | |  | *e_1,2_* |  |
| *sAA_1,3_* |  | 1 | *sAA_1,2_* | *sAA_1,1_* | *11* | *121* |  |  |  | | *e_1,3_* |  |
| *sAA_1,4_* |  | 1 | *sAA_1,3_* | *sAA_1,2_* | *12* | *144* |  |  | |  | *e_1,4_* |  |
| *sAA_1,5_* |  | 1 | *sAA_1,4_* | *sAA_1,3_* | *13* | *169* |  |  | |  | *e_1,5_* |  |
| *-* |  | - | *sAA_1,5_* | *sAA_1,4_* |  |  |  |  | |  | - |  |
| *-* |  | - | - | *sAA_1,5_* |  |  |  |  | |  | - |  |
| *sAA_2,1_* |  | 1 | - | ***-*** | *9* | *81* |  |  | |  | *e_2,1_* |  |
| *sAA_2,2_* |  | 1 | *sAA_2,1_* | *-* | *10* | *100* |  | *b_0_* | |  | *e_2,2_* |  |
| *sAA_2,3_* | = | 1 | *sAA_2,2_* | *sAA_2,1_* | *11* | *121* |  | *b_1_* | |  | *e_2,3_* |  |
| *sAA_2,4_* |  | 1 | *sAA_2,3_* | *sAA_2,2_* | *12* | *144* | * | *b_2_* | | + | *e_2,4_* |  |
| *sAA_2,5_* |  | 1 | *sAA_2,4_* | *sAA_2,3_* | *13* | *169* |  | *b_3_* | |  | *e_2,5_* |  |
| ***-*** |  | - | *sAA_2,5_* | *sAA_2,4_* |  |  |  | *b_4_* | |  | - |  |
| ***-*** |  | - | - | *sAA_2,5_* |  |  |  |  | |  | - |  |
| *sAA_3,1_* |  | 1 | - | - | *9* | *81* |  |  | |  | *e_3,1_* |  |
| *sAA_3,2_* |  | 1 | *sAA_3,1_* | - | *10* | *100* |  |  | |  | *e_3,2_* |  |
| *sAA_3,3_* |  | 1 | *sAA_3,2_* | *sAA_3,1_* | *11* | *121* |  |  | |  | *e_3,3_* |  |
| *sAA_3,4_* |  | 1 | *sAA_3,3_* | *sAA_3,2_* | *12* | *144* |  |  | |  | *e_3,4_* |  |
| *sAA_3,5_* |  | 1 | *sAA_3,4_* | *sAA_3,3_* | *13* | *169* |  |  | |  | *e_3,5_* |  |
| *-* |  | - | *sAA_3,5_* | *sAA_3,4_* |  |  |  |  | |  | - |  |
| *-* |  | - | - | *sAA_3,5_* |  |  |  |  | |  | - |  |

**SD Table.**

**Table D. How to enter the data for analysis in Stata, Panel Data module**

| ***sAA_P1_*** | ***sAA_P2_*** | ***sAA_P3_*** | ***h*** | ***h^2^*** |
| --- | --- | --- | --- | --- |
| *sAA_1,1_* | *sAA_2,1_* | *sAA_3,1_* | *9* | *81* |
| *sAA_1,2_* | *sAA_2,2_* | *sAA_3,2_* | *10* | *100* |
| *sAA_1,3_* | *sAA_2,3_* | *sAA_3,3_* | *11* | *121* |
| *sAA_1,4_* | *sAA_2,4_* | *sAA_3,4_* | *12* | *144* |
| *sAA_1,5_* | *sAA_2,5_* | *sAA_3,5_* | *13* | *169* |
