# Supplementary material for: Intensive longitudinal modelling predicts diurnal activity of salivary alpha-amylase
Source: PLoS One. 2019 Jan 23;14(1):e0209475. doi: 10.1371/journal.pone.0209475 (PMC6343885; doi:10.1371/journal.pone.0209475)
Supplement: S1 Table — The means and standard deviations are added for each participant (sAAj,■ and sj,■) and for each time (sAA■,t and s■,t). a Values of 0 mean that the original value of the salivary variable is 1 (ln(1) = 0). (DOCX) [file pone.0209475.s002.docx]

| Hour | | | | | | | | | | | | | | | |
| --- | --- | --- | --- | --- | --- | --- | --- | --- | --- | --- | --- | --- | --- | --- | --- |
| Participant | 09:00 | 10:00 | 11:00 | 12:00 | 13:00 | 14:00 | 15:00 | 16:00 | 17:00 | 18:00 | 19:00 | 20:00 | 21:00 | ${sAA}_{j,.}$ | $\text{ s}_{j\text{,}.}$ |
| 1 | 3.18 | 3.66 | 4.38 | 4.69 | 4.91 | 4.53 | 3.91 | 4.80 | 4.83 | 4.57 | 4.83 | 4.86 | 5.31 | 4.50 | .59 |
| 2 | 2.48 | 3.56 | 4.08 | 3.69 | 3.09 | 3.89 | 4.11 | 4.49 | 4.39 | 4.48 | 4.39 | 3.89 | 3.99 | 3.89 | .58 |
| 3 | 0.69 | .00 ^a^ | .00 ^a^ | 1.39 | 1.39 | .69 | 1.39 | 1.10 | 1.39 | 1.95 | 1.39 | 1.79 | 2.08 | 1.17 | .66 |
| 4 | 3.04 | 3.50 | 3.69 | 3.85 | 4.42 | 4.67 | 4.65 | 5.38 | 5.16 | 4.30 | 5.16 | 5.20 | 4.69 | 4.44 | .73 |
| 5 | 3.64 | 3.69 | 4.66 | 4.92 | 4.74 | 4.11 | 4.34 | 4.39 | 4.32 | 4.67 | 4.32 | 4.84 | 3.47 | 4.32 | .47 |
| 6 | 1.95 | 1.95 | 3.14 | 2.94 | 2.64 | 2.89 | 3.26 | 2.64 | 2.77 | 3.40 | 2.77 | 3.83 | 4.33 | 2.96 | .66 |
| 7 | 2.83 | 3.00 | 3.00 | 2.77 | 2.83 | 3.00 | 3.30 | 3.58 | 3.22 | 3.43 | 3.22 | 3.18 | 3.00 | 3.10 | .24 |
| 8 | 3.66 | 4.49 | 3.89 | 3.76 | 2.94 | 4.37 | 4.84 | 5.27 | 3.95 | 4.30 | 3.95 | 4.38 | 4.06 | 4.15 | .58 |
| 9 | 1.39 | 1.61 | 2.94 | 2.56 | 3.33 | 3.22 | 3.33 | 2.48 | 2.20 | 2.66 | 2.20 | 2.68 | 2.79 | 2.57 | .61 |
| 10 | 3.69 | 3.18 | 3.91 | 3.26 | 4.04 | 3.53 | 4.29 | 4.41 | 3.83 | 3.91 | 3.83 | 4.47 | 4.55 | 3.91 | .44 |
| 11 | 2.20 | 2.89 | 2.77 | 3.40 | 3.26 | 2.30 | 3.53 | 4.22 | 3.99 | 4.64 | 3.99 | 4.16 | 4.16 | 3.50 | .78 |
| 12 | 3.40 | 4.55 | 5.25 | 5.63 | 5.14 | 5.57 | 5.81 | 5.78 | 5.75 | 5.07 | 5.75 | 4.97 | 5.21 | 5.22 | .67 |
| 13 | 2.30 | 3.09 | 2.48 | 3.37 | 2.89 | 4.28 | 3.95 | 3.47 | 4.23 | 2.83 | 4.23 | 4.74 | 5.32 | 3.63 | .91 |
| 14 | 3.64 | 3.66 | 4.30 | 3.37 | 4.19 | 3.58 | 3.93 | 4.81 | 4.54 | 5.00 | 4.54 | 5.42 | 4.94 | 4.30 | .64 |
| 15 | 3.47 | 2.20 | 2.48 | 3.50 | 2.20 | 3.50 | 3.33 | 3.04 | 3.45 | 3.00 | 3.37 | 2.30 | -- | 2.99 | .54 |
| 16 | 2.40 | 2.20 | 2.08 | 2.20 | 2.83 | 2.56 | 2.48 | 2.83 | 2.89 | 3.09 | 2.89 | 2.64 | 3.09 | 2.63 | .34 |
| 17 | 2.48 | 3.53 | 4.17 | 4.16 | 4.28 | 4.03 | 4.37 | 4.55 | 4.50 | 4.72 | 4.50 | 4.93 | 4.67 | 4.22 | .63 |
| 18 | 3.64 | 3.37 | 4.84 | 4.39 | 4.85 | 3.87 | 5.42 | 4.84 | 5.91 | 5.41 | 5.91 | 5.00 | 5.09 | 4.81 | .81 |
| 19 | 4.29 | 4.74 | 4.74 | 4.36 | 4.23 | 4.34 | 4.65 | 5.12 | 5.24 | 4.96 | 5.24 | 5.15 | 5.00 | 4.78 | .37 |
| ${sAA}_{.,t}$ | 2.86 | 3.10 | 3.52 | 3.59 | 3.59 | 3.63 | 3.94 | 4.06 | 4.03 | 4.02 | 4.03 | 4.13 | 4.21 |  |  |
| $\text{ s}_{\text{.}\text{,t}}$ | .91 | 1.13 | 1.24 | 1.00 | 1.04 | 1.06 | 1.01 | 1.20 | 1.18 | .97 | 1.18 | 1.10 | .97 |  |  |
