# Supplementary material for: Intensive longitudinal modelling predicts diurnal activity of salivary alpha-amylase
Source: PLoS One. 2019 Jan 23;14(1):e0209475. doi: 10.1371/journal.pone.0209475 (PMC6343885; doi:10.1371/journal.pone.0209475)
Supplement: S2 Table — DV: sAAj,t, IVs: hr, hr2. (DOCX) [file pone.0209475.s003.docx]

| **A. Information Criteria** | |
| --- | --- |
| -2 Log Likelihood | 399.226 |
| Akaike's Information Criterion (AIC) | 409.226 |
| Hurvich and Tsai's Criterion (AICC) | 409.476 |
| Bozdogan's Criterion (CAIC) | 431.752 |
| Schwarz's Bayesian Criterion (BIC) | 426.752 |

| **B. Estimates of Fixed Effects** | | | | | | | |
| --- | --- | --- | --- | --- | --- | --- | --- |
| Parameter | Estimate | Std. Error | df | t | p | 95% Confidence Interval | |
|  |  |  |  |  |  | Lower  Bound | Upper Bound |
| *Intercept* | .157 | .566 | 229 | .278 | .781 | -.957 | 1.271 |
| *hr* | .400 | .073 | 227 | 5.508 | <.001 | .257 | .543 |
| *hr^2^* | -.010 | .002 | 227 | -4.195 | <.001 | -.015 | -.005 |

| **C. Estimates of Covariance Parameters** | | | | | | | |
| --- | --- | --- | --- | --- | --- | --- | --- |
| Parameter | | Estimate | Std. Error | Wald Z | p | 95% Confidence Interval | |
|  |  |  |  |  |  | Lower Bound | Upper Bound |
| $s_{e}^{2}$ (level 1 Var) | | .218 | .020 | 10.654 | <.001 | .181 | .261 |
| $s_{u}^{2}$(level 2 Var) |  | .897 | .297 | 3.025 | .002 | .469 | 1.714 |
