# Supplementary material for: Intensive longitudinal modelling predicts diurnal activity of salivary alpha-amylase
Source: PLoS One. 2019 Jan 23;14(1):e0209475. doi: 10.1371/journal.pone.0209475 (PMC6343885; doi:10.1371/journal.pone.0209475)
Supplement: S3 Table — DV: sAAj.t, IVs: sAAj,t-1, sAAj,t-2. (DOCX) [file pone.0209475.s004.docx]

| **A. Information Criteria** | |
| --- | --- |
| -2 Log Likelihood | 308.000 |
| Akaike's Information Criterion (AIC) | 316.000 |
| Hurvich and Tsai's Criterion (AICC) | 316.197 |
| Bozdogan's Criterion (CAIC) | 333.350 |
| Schwarz's Bayesian Criterion (BIC) | 329.350 |

| **B. Estimates of Fixed Effects** | | | | | | | |
| --- | --- | --- | --- | --- | --- | --- | --- |
| Parameter | Estimate | Std. Error | df | t | p | 95% Confidence Interval | |
|  |  |  |  |  |  | Lower Bound | Upper Bound |
| *Intercept* | .562 | .126 | 208 | 4.441 | <.001 | .312 | .811 |
| *sAA_j.t-1_* | .523 | .063 | 208 | 8.349 | <.001 | .399 | .646 |
| *sAA_j.t-2_* | .365 | .062 | 208 | 5.904 | <.001 | .243 | .487 |

| **C. Estimates of Covariance Parameters** | | | | | | |
| --- | --- | --- | --- | --- | --- | --- |
| Parameter | Estimate | Std. Error | Wald Z | p | 95% Confidence Interval | |
|  |  |  |  |  | Lower Bound | Upper Bound |
| $s_{e}^{2}$ | .257 | .025 | 10.198 | <.001 | .212 | .312 |
|  | | | | | | |
