# Supplementary material for: Intensive longitudinal modelling predicts diurnal activity of salivary alpha-amylase
Source: PLoS One. 2019 Jan 23;14(1):e0209475. doi: 10.1371/journal.pone.0209475 (PMC6343885; doi:10.1371/journal.pone.0209475)
Supplement: S4 Table — (DOCX) [file pone.0209475.s005.docx]

| **A. Information Criteria** |  |
| --- | --- |
| -2 Log Likelihood | 303.008 |
| Akaike's Information Criterion (AIC) | 317.008 |
| Hurvich and Tsai's Criterion (AICC) | 317.568 |
| Bozdogan's Criterion (CAIC) | 347.371 |
| Schwarz's Bayesian Criterion (BIC) | 340.371 |

| **B. Estimates of Fixed Effects** | | | | | | | |
| --- | --- | --- | --- | --- | --- | --- | --- |
| Parameter | Estimate | Std. Error | df | t | p | 95% Confidence Interval | |
|  |  |  |  |  |  | Lower  Bound | Upper Bound |
| *Intercept* | 2.021 | .992 | 208 | 2.038 | .043 | .065 | 3.977 |
| *hr* | -.176 | .128 | 208 | -1.378 | .170 | -.429 | .076 |
| *hr^2^* | .004 | .003 | 208 | 1.211 | .227 | -.003 | .012 |
| *sAA_j.t-1_* | .516 | .061 | 208 | 8.338 | <.001 | .394 | .638 |
| *sAA_j.t-2_* | .394 | .062 | 208 | 6.310 | <.001 | .271 | .517 |

| **C. Estimates of Covariance Parameters** | | | | | | |
| --- | --- | --- | --- | --- | --- | --- |
| Parameter | Estimate | Std. Error | Wald Z | p | 95% Confidence Interval | |
|  |  |  |  |  | Lower Bound | Upper Bound |
| $s_{e}^{2}$ | .251 | .024 | 10.198 | <.001 | .207 | .304 |
| $s_{u}^{2}$ | .000^a^ | .000 | . | . | . | . |

^a^ This parameter of covariance is redundant. The test statistic and the confidence interval can not be calculated.
